# The End of a Noble Career

**Published:** 1899-06

**Authors:** 


### THE END OF A NOBLE CAREER.

PEACEFULLY passing away among those with whom his whole life had been spent, surrounded by those near and most dear to him, ending a life well lived, closing a noble career, fulfilling more than the allotted years given man to live, there went out from our midst our esteemed and honored colleague, Dr. Isaiah Michener.

For quite sixty years his quiet influence, his patient work, his untiring devotion, brought to him in the evening of his life the grateful realization of a more advanced, a higher, and a rapidly progressing profession. Loving his calling as he did, pursuing its toil and hardships under the gravest difficulties and most trying circumstances, without a murmur, with no complaint of the dim light that faintly illumined the way of veterinary science, he made most of all his opportunities and well used every avenue of advancement opened to him, and left much for us all to be thankful and appreciative of as a profession—all that one can hope to earn or deserve in his own community, that which is best of all—a *good name.*

As the threads of life spun out the shadows of advancing years grew darker and the ebb tide nearly run, with many of the infirmities of old age, he lost none of his interest in his chosen profession, and was thrice proud of its progress and achievements.

Such, in brief, are a few of the characteristics that marked the life of our deceased colleague. But to few of us will it be given to labor so many years in professional work; but should we be half so faithful in maintaining its honor, advancing its progress, shedding the light of a studious career, giving only the best we have to those who entrust to our keeping their valued and valuable stock, we will earn a "well done, thou good and faithful servant."

---

The Chicago veterinarians are again trying to induce Mayor Harrison to employ competent veterinarians in the police and fire departments.

